# Vitamin D and Neurological Diseases: An Endocrine View

**DOI:** 10.3390/ijms18112482

**Published:** 2017-11-21

**Authors:** Carolina Di Somma, Elisabetta Scarano, Luigi Barrea, Volha V. Zhukouskaya, Silvia Savastano, Chiara Mele, Massimo Scacchi, Gianluca Aimaretti, Annamaria Colao, Paolo Marzullo

**Affiliations:** 1 IRCCS SDN, Napoli Via Gianturco 113, 80143 Naples, Italy; cdisomma@unina.it; 2 Dipartimento di Medicina Clinica e Chirurgia, Divisione di Endocrinologia Università “Federico II” Napoli, 80138 Napoli, Italy; el.scarano@gmail.com (E.S.); volha.zhukouskaya@gmail.com (V.V.Z.); sisavast@unina.it (S.S.); colao@unina.it (A.C.); 3 I.O.S. & COLEMAN Srl, 80011 Napoli, Italy; luigi.barrea@unina.it; 4 Division of General Medicine, IRCCS Istituto Auxologico Italiano, Ospedale S. Giuseppe, Via Cadorna 90, 28824 Piancavallo VB, Italy; chiara.mele1989@gmail.com (C.M.); massimo.scacchi@unimi.it (M.S.); 5 Department of Translational Medicine, University of Piemonte Orientale, Via Solaroli 17, 28100 Novara, Italy; gianluca.aimaretti@med.uniupo.it; 6 Department of Clinical Sciences and Community Health, Università di Milano, 20122 Milano, Italy

**Keywords:** vitamin D, neurological disease, biomarker, hormones

## Abstract

Vitamin D system comprises hormone precursors, active metabolites, carriers, enzymes, and receptors involved in genomic and non-genomic effects. In addition to classical bone-related effects, this system has also been shown to activate multiple molecular mediators and elicit many physiological functions. In vitro and in vivo studies have, in fact, increasingly focused on the “non-calcemic” actions of vitamin D, which are associated with the maintenance of glucose homeostasis, cardiovascular morbidity, autoimmunity, inflammation, and cancer. In parallel, growing evidence has recognized that a multimodal association links vitamin D system to brain development, functions and diseases. With vitamin D deficiency reaching epidemic proportions worldwide, there is now concern that optimal levels of vitamin D in the bloodstream are also necessary to preserve the neurological development and protect the adult brain. The aim of this review is to highlight the relationship between vitamin D and neurological diseases.

## 1. Introduction

The vitamin D system comprises steroid pro-hormones, their metabolites, carriers and enzymes involved in vitamin D metabolism [1]. Vitamin D occurs in nature as two main forms: vitamin D2 (ergocalciferol), which is photochemically synthesized in plants, and vitamin D3 (cholecalciferol), which is synthesized in the skin after exposure to sunlight, in particular ultraviolet B radiation at an appropriate wavelength of 280–320 nm. Vitamin D metabolism is complex and tightly regulated [1]. The classical synthetic vitamin D pathway involves sequential 25-hydroxylation [25(OH)D] and 1α-hydroxylation [1,25(OH)2D] of vitamin D2 and D3 precursors in the liver and kidney, respectively. Vitamin D metabolites circulate in the bloodstream as bound to the vitamin D binding protein (DBP). Vitamin D catabolism is mostly mediated by 24-hydroxylase (CYP24A1), which converts 1,25(OH)2D (calcitriol) in 1,24,25(OH)3D. This catabolite binds with significantly lower affinity the vitamin D receptor (VDR), and is further processed into excretion products (calcitroic acid). Classically, vitamin D has a recognized role in the regulation of bone health physiology and calcium-phosphorus homeostasis, by acting at the level of the skeletal bone, intestine and kidney. There is now consistent evidence showing that several “non-calcemic” effects of vitamin D metabolites occur in vitro and in vivo, and increasing consideration is given to vitamin D status as a marker of general health, since low vitamin D levels are associated with the development and progress of autoimmunity, infectious diseases, diabetes mellitus, cardio-metabolic disorders, obesity, neuromuscular disorders, and cancer [2]. Vitamin D and its congeners yield both genomic and non-genomic actions. Genomic actions are mediated by the VDR, a member of the steroid hormone superfamily. The VDR is a nuclear receptor present in over 30 human tissues, which regulates approximately 3% of the human genome (i.e., about 700 genes) [3,4,5]. Nuclear VDRs are present in the majority of cells of the body and form the basis for the investigations in the extra-skeletal benefits of vitamin D.

VDR, as well as 25-hydroxylase and 1α-hydroxylase, the enzymes controlling vitamin D activation, along with 24-CYP24A1, the enzyme controlling vitamin D degradation, are expressed in the brain [6,7,8]. The main consequence is that the central nervous system (CNS) is able to synthesize its own vitamin D, which yields auto- or paracrine neurosteroid actions at the local level [8]. Neurons and glial cells, particularly in the temporal, cingulate, and orbital cortices and in the thalamus, nucleus accumbens, stria terminalis, and amygdala, express the VDR and 1α-hydroxylase [9]. The distribution of the VDR and 1α-hydroxylase has also been investigated in the adult human brain, and is similar to that found in the rat [8]. Calcitriol-ligand binding to VDR allows heterodimerization with retinoid X receptor (RXR) and its ligand (9-*cis*-retinoic acid), then the VDR/RXR complex binds specific sequences in the promoter region of vitamin D responsive genes (vitamin D response elements; VDREs). Vitamin D actions on brain development involve effects on cellular proliferation, differentiation, calcium signalling, neurotrophism and neuroprotection. It also appears that vitamin D plays a role in neurotransmission and synaptic plasticity, and a link has been described between vitamin D and dopaminergic neurotransmission [10,11,12]. Collectively, the physiological effects of vitamin D in brain functions include the promotion of neurotransmission, neurogenesis, synaptogenesis, amyloid clearance and the prevention of neuronal death. It is, thus, not surprising that observational studies have documented associations between higher serum vitamin D concentrations and healthier cognitive performance [13].

Another recent field of interest regards VDR gene polymorphisms. Preliminary data suggest that single nucleotide polymorphisms (SNPs) in the VDR gene may have roles in the development of multiple sclerosis, Parkinson’s disease, and Alzheimer’s disease [14,15]. However, recent results on the association between VDR gene polymorphisms and different neurological diseases are somewhat contradictory, and the role of VDR in the aetiology of neurological diseases is still uncertain. Further investigations are needed to obtain more definitive results. 

More than 85% of circulating 25(OH)D and 1,25(OH)2D, the bioactive metabolite of vitamin D, are tightly bound to the DBP, which circulates in molar excess with respects to its ligands. As little as 5% of all circulating DBP is bound to vitamin D metabolites. DBP can also bind globular actin (G-actin) with high affinity and sequesters G-actin released into the circulation upon tissue/cell damage or necrosis, thus participating to the organism’s actin scavenging system [16]. This peculiar action confers DBP a vital role, since circulating free G-actin polymerization into long filaments can initiate disseminated intravascular coagulation (DIC) if not rapidly cleared. DBP also intervenes to regulate the chemotactic activity of complement 5a (C5a), and it partakes in the inflammatory cascade as a precursor of macrophage activation factor (MAF), which originates from DBP through modifications of the glycosylated residues [17]. The “free hormone hypothesis” infers that DBP works as a reservoir and delivery system for free vitamin D metabolites to target tissues at the cellular level [17]. The free hormone hypothesis states that the biological activity of a given hormone is affected by its unbound, rather than protein-bound, concentration in the plasma. It has been suggested that the free hormone hypothesis could exist occur even if tissue uptake is caused by a mechanism involving one or more circulating protein-bound pools of hormone, as well as for the hydroxylated metabolites of vitamin D. Nevertheless, endocytic receptors have also been identified which are capable of transporting DBP-vitamin D complexes inside the target cells [18], and are reckoned as essential for the renal metabolism of vitamin D. Of note, DBP-dependent transport mechanisms are also thought to contribute to vitamin D access to the CNS [19]. Given the multifaceted activities of DBP, an alteration of its circulating levels may impact pathophysiology in different ways, one of which could imply modifications of vitamin D bioavailability.

Non-genomic actions of vitamin D have been discovered in many systems, and only recently they have been identified in the brain [20]. Non-genomic pathways cooperate with the classical genomic pathway to *trans*-activate the VDR and exert the effects of calcitriol. Non-genomic signalling is rapid, does not depend on transcription and may indirectly affect transcription via cross-talk with other signalling pathway. Data suggest that non-genomic actions of vitamin D occur at the plasma membrane level and involve a non-classical membrane-associated receptor, and a calcitriol-membrane-associated rapid response steroid binding protein (1,25-MARRS) [21,22]. Also, non-genomic actions of calcitriol induce calcium translocation across intestinal membranes, and calcitriol binding to membrane receptor activates signalling cascades leading to an increase in intracellular calcium flux via opening of voltage-gated calcium channels. This may, in turn, activate other growth regulatory pathways (e.g., rat sarcoma family of GTPases (RAS), murine leukemia viral oncogene (Raf)-1, mitogen-activated protein kinases/extracellular signal-regulated kinases (MAPK/ERK), as described in skeletal muscle cells). Moreover, ERK can enhance transcriptional activity of the VDR and non-genomic activation of protein kinase C (PKC) may stabilize VDR via phosphorylation [23,24,25]. There is also evidence that 1,25(OH)2D modulates l-type calcium channel (LTCC) functions, and these effects can influence neuronal function [26], such as neuronal maturation during developmental stages and/or neuroprotection during adulthood [27,28]. 

**Figure 1:**
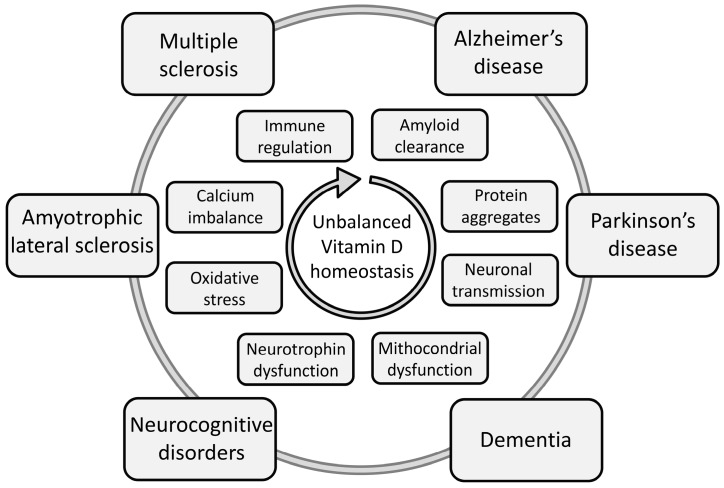
Schematic overview of neurological disorders and their underlying mechanisms relating to impaired homeostasis of the vitamin D system.

As summarized in Figure 1, vitamin D deficiency has been mechanistically and clinically linked to neurological diseases and neuropsychological disorders, cognitive impairment and neurodegenerative diseases [20,29,30,31,32,33,34]. While vitamin D deficiency may act as a common risk factor [29], it should be borne in mind that the origin of these disorders is often complex and involves both genetic and environmental causes. Interestingly, animal and human data have suggested that vitamin D status, particularly vitamin D deficiency, may intervene during adulthood to modulate the exacerbation of inherent brain disorders and/or impair the recovery from brain stressors [29]. It should be mentioned here that vitamin D deficiency has also been related to mood disorders and a number of psychiatric conditions. While some cross-sectional and epidemiologic studies have found that low levels of vitamin D are significantly associated with higher levels of depressive symptoms or with a depression diagnosis, data are currently argued for the lack of causality, that is, current evidence does not definitively demonstrate that vitamin D deficiency is a cause of or risk for developing depression nor that vitamin D is an effective therapy for depression. For these reasons, we reckon that this topic should be distinctly addressed in further reviews.

One key aspect related to vitamin D homeostasis and neurological disorders is the role of vitamin D supplementation in 25(OH)D deficient patients. Results of clinical trials conducted in patients with neurological disorders have, so far, provided controversial findings [20,34,35,36,37,38,39], and the potential confounding effect of baseline vitamin D status and supplementation doses exists. It is of note that interventional studies conducted in the general population treated with vitamin D have mostly focused on the relationship between achieved vitamin D levels and the prevention of falls and fractures [40,41,42,43]. At present, the Institute of Medicine [44] recommends attaining 25(OH)D concentrations ranging between 20 and 50 ng/mL during vitamin D supplementation, whereas the Endocrine Society, the International Osteoporosis Foundation, and the American Geriatric Society suggest that a minimum level of 30 ng/mL is needed to minimize the risk of falls and fracture in older adults [45,46,47]. For concentration purposes: to convert ng/mL to nmol/L: 1 ng/mL = 2.5 nmol/L; for dosing purposes: to convert mcg to IU: mcg/0.025 = IU. The Institute of Medicine systematic review also voiced concern for serum 25(OH)D concentrations above 50 ng/mL [44]. These concerns were based on an increased risk of fractures and certain types of cancer (pancreatic and prostate) in people receiving high dose vitamin D supplementation [43]. In general, vitamin D should be supplemented to reach 25(OH)D level of at least 20 ng/mL, and a level of 30 ng/mL is recommended by most guidelines [45,46,47]. A critical 25(OH)D threshold of 200 ng/mL should be mandatorily avoided to reduce the risk of hypercalcemia and symptoms of vitamin D excess. Expectedly, the required dose of vitamin D supplements varies between individuals depending on baseline 25(OH)D level, seasonality, latitude, ethnicity, nutrition, adiposity, dosing and type of vitamin D analog used for supplementation [48]. The optimal approach for supplementation in the general population, and in patients with neurological diseases, has not been established yet.

For many reasons, therefore, vitamin D supplementation in neurological diseases needs to be optimized in terms of dosing and timing to generate a therapeutic potential. Recent studies suggest that vitamin D supplementation doses should be individualized depending on baseline vitamin D status and responsiveness. The potentially interfering activity of factors such as adiposity, enzymes catalyzing the different pathways [20,29,31] and VDR genotypes should be appropriately addressed [49]. Large, randomized, well-controlled clinical trials are also awaited to provide evidence on the safety and tolerability of vitamin D as adjunct therapy. The aim of this review is to provide an unbiased overview on the relationship between vitamin D and neurological diseases.

## 2. Multiple Sclerosis and Vitamin D

Multiple sclerosis (MS) is a debilitating slow progressive disorder of the central nervous system, which is characterized by axonal injury and demyelination in the spinal cord and brain. Although its etiology is unclear, it seems to be multifactorial, with dysregulation of the immune response, environmental factors and genetic determinants all playing a contributive role [50]. In fact, genetic, epidemiological and immunological studies showed that MS is an autoimmune disease [51,52] influenced by environmental risk factors such as infections, cigarette smoking, obesity, and inadequate serum levels of vitamin D and/or its metabolites [53,54,55,56]. Attention has been recently given to the study of the microbiome in MS and its relationship with environmental stressors [50,57]. Emerging cross-sectional, case-control studies have shown that microbiota composition differs between MS subjects and controls [58,59]. Nevertheless, human studies supporting the role of microbiota in MS are still scarce.

Of the environmental factors identified in association to MS risk and capable of influencing its clinical course, vitamin D is among the strongest and most consistently found in connection [60,61,62,63,64]. A direct relationship has been observed between latitude and the prevalence of MS, which suggests a role for UV radiation and vitamin D in MS development [65]. Other studies showed that the risk of MS decreases with increasing intake of vitamin D [66], and serum 25(OH)D levels are significantly lower in patients with MS as compared to healthy controls [67]. Genetic studies found a relationship between MS susceptibility and SNPs of enzymes genes relating to vitamin D metabolism, namely, CYP27B1 and CYP24A1 [68,69]. Genome-wide association studies have identified more than 100 non-human leucocyte antigen (HLA) genetic risk loci, many acting as cooperative networks. However, each of these individual loci exerts modest influence on MS risk, and major histocompatibility complex (MHC) remains the key susceptibility locus [70]. Other studies also focused on VDR gene polymorphisms in association with increased susceptibility to develop MS, or with modulation and progression of MS. As such, significant associations were obtained for the VDR gene polymorphisms Bsm-I in a Japanese cohort, Apa-I in Japanese and Australian cohorts, and Taq-I in an Australian cohort [71,72]. Further, a trend for the polymorphism Fok-I was described in a UK cohort study [73], while a Canadian study found no preferential transmission of Apa-I and Taq-1 from MS-affected parents to affected offspring, and no association between Taq-I polymorphism and MS was found in another UK study [73,74]. Genetic data also suggest that vitamin D may act on the risk of MS by influencing the regulation of vitamin D-responsive genes involved in immunity, such as the HLA-DRB1* 1501 allele, which has a highly conserved VDR responsive element in its promoter [70,75]. 

Vitamin D influences the cytokine profile and the inflammasome [76]. In vitro studies support the evidence that vitamin D prevents interleukin (IL) 2, IL12 and interferon-gamma production, as well as B cells production [76,77,78]. In addition, vitamin D negatively regulates TH17-mediated autoimmune diseases, one example of which is MS [79]. Activation of the VDR by vitamin D stimulates a shift from proinflammatory Th1 responses to anti-inflammatory Th2 responses in brain [76]. Proliferation suppression assays showed an association between high 25(OH)D levels and improved regulatory T cell function in patients with MS [80,81]. Interestingly, while the Epstein-Barr virus (EBV) infection appears to be a necessary (but insufficient) condition for adult MS to develop [82], low vitamin D could act on the immune response to EBV to increase the risk of MS [83,84,85]. Therefore, a role for vitamin D in the immune regulation of MS is, for many reasons, biologically plausible. 

From a clinical viewpoint, several observations suggest a role for vitamin D in MS. Evidence relating MS to impaired vitamin D status is prompted by studies conducted both in children and adults from Australia [86], United States [63,64,87,88,89] and Europe [90]. Common associations involve circulating 25(OH)D levels, geographic distribution of MS, bone mass density in MS patients, seasonal fluctuations of 25(OH)D, parameters of MS disease, MS births, MS course in pregnancy, and results of genetic analysis on the VDR. As such, a matched case-control study using neonatal dried blood spots samples from 521 patients with MS outlined an association between 25(OH)D levels and MS risk as evaluated by odds ratios (ORs), showing a higher MS risk in individuals in the bottom 25(OH)D quintile compared to the top quintile (OR, 0.53), whereas a 25 nmol/L increase in neonatal 25(OH)D resulted in a 30% reduced risk of MS (OR, 0.70) [91]. In a Finnish study on vitamin D levels in over 1000 pregnant women who were later diagnosed with MS and over 2000 comparable women without MS, a two-fold increased MS risk was found among vitamin D–deficient women compared with vitamin D-replete women, and each 50 nmol/L increase in vitamin D level was associated with a 39% lower risk for MS [92]. Inversely, other studies failed to demonstrate direct associations between MS and vitamin D, whereas one study found low 25(OH)D in male but not female patients with MS [93,94,95]. 

Sun exposure is, indeed, the most important predictor of vitamin D status. In retrospective studies, sun exposure during childhood and adolescence was associated with a lower risk of adult-onset MS [96,97,98,99], although these surveys are potentially challenged by the accuracy of recall. Studies in individuals migrating from the tropics to temperate regions before or during adolescence also found an increased risk of MS [100]. Thus, it could be hypothesized that low sun exposure at young ages could impair vitamin D status, hence increase the risk of MS later in life. However, scant data on the link between 25(OH)D and MS exist in world regions where MS is extremely rare, such as peri-equatorial countries, Africa, and a few Asian regions. Evidence of vitamin D insufficiency in the rare cases of MS diagnosed in these regions would give strength to the hypothetical association between vitamin D status and MS.

Brain imaging studies have shown that vitamin D influences the disease activity in patients with MS and relapse risk [61]. In relapsing-remitting MS (RRMS), the severity of vitamin D insufficiency was related to higher levels of disability measured by the Expanded Disability Status Scale (EDSS), and was greater in patients with progressive forms of MS as compared with RRMS, suggesting that vitamin D status could have a prognostic value in MS [86,101]. However, this link was not confirmed in other studies including fewer patients [102,103].

It is also interesting to reckon that some disproportion has been noticed in the female/male ratio of MS patients with relapse-onset disease (relapsing remitting (RR) and secondary progressive (SP)). An association between skin type and MS-related disability has only been documented in female patients [104], implying a gender-related effect in vitamin D metabolism, while a rodent study found an association between dietary vitamin D and inhibition of severe experimental autoimmune encephalomyelitis (EAE) in female but not male mice [105], suggesting a gender-based difference in vitamin D responsiveness. Oppositely, a recent prospective study on 101 patients and 107 controls followed during one year with 25(OH)D and 1,25(OH)2D measurements in summer and winter [106] documented no difference in vitamin D levels between groups, yet an association between high levels of 25(OH)D and lower incidence of MS and MS-related disability could only be observed in the female population. However, the processes relating to clinical manifestations of MS, such as inflammation, demyelination, axonal damage and repair mechanisms, is unequally distributed across patients and genders, therefore suggesting the intervention of interacting factors relating to the disease and response to treatments [107]. 

Despite the extensive literature focusing on cerebro-spinal fluid (CSF) biomarkers in MS, only qualitative and quantitative biochemical methods have been used to assess intrathecal production of immunoglobulins [108]. Considering the multiple roles of DBP, which include actin sequestration and a range of less-defined roles in modulating immune and inflammatory responses [16,109], several studies have suggested a potential modulatory role for DBP in the development of MS [110,111,112,113,114,115], although DBP results in CSF or plasma have generated discrepant results [110,113,116,117,118,119,120]. Recently, Perga et al. proposed a novel biomolecular tool consisting of two isoforms of DBP and ApoE in CSF, which may aid monitoring the progression of MS [121]. If validated in a larger population, this tool could provide insights on clinical management, treatment strategy and long-term prognosis of MS patients. Likewise, studies on DBP glycosylation and the activity of the enzymes involved in glycosylation/deglycosylation could assist researchers in deciphering the pathogenetic pathways associated with the onset, progression and response to treatments in MS patients.

There are studies suggesting that vitamin D supplementation could be used therapeutically for subjects with MS or those at a risk for MS. The optimal approach for vitamin D supplementation in the general population, and in patients with MS, has not been convincingly established, with controversies relating to dose, time and target levels of vitamin D treatment [44,45,46,47]. The relationship between target vitamin D levels and modulation of the risk/progression of MS may differ from that used for skeletal health [122], and vitamin D supplementation trials in MS have shown inconclusive outcome results [31] due to the lack of long-term follow-up and methodological bias. Preliminary studies inferred that high-dose vitamin D supplementation is generally well tolerated in MS patients. Phase I and phase II trials using high-dose vitamin D3 supplementation have shown that patients with MS can tolerate doses as high as 20,000 IU daily for 12 weeks [123], as well as escalating doses of vitamin D3 up to 40,000 IU daily over 28 weeks [124], with barely detectable side effects. However, findings obtained in short-term studies await more definitive evidence to prove the safety of long-term, high-dose vitamin D supplementation. Longer phase II or phase III randomized clinical trials (RCTs) are ongoing and will provide more evidence regarding the safety and tolerability of vitamin D as an adjunct therapy [125,126,127]. In a recent review, Pierrot-Deseilligny et al., [128] suggested a pragmatic and practical approach for vitamin D3 supplementation using moderate oral doses (between 2000 and 4000 IU/day) for all types of MS patients, including pregnant women [129]. These authors underlined the advantages of supplementation doses: (1) the correction of vitamin D insufficiency existing in the great majority of MS patients, with 25(OH)D serum levels thus increasing up to the currently recommended range (30–60 ng/mL) [130]; (2) the need to prevent osteoporosis, attenuate infections, as well as to improve non classical clinical outcomes [131] in patients with marked vitamin D deficiency; (3) the safety of a moderate supplementation in terms of hypercalcemia and other significant adverse events [130,132]; (4) the control of inflammatory components of the disease. In temperate countries, the supplementation should never be stopped since there is no durable storage of this vitamin in the organism. However, analysis of vitamin D and its metabolites should be made prior to supplementation so as to monitor responsiveness to treatment at preset times after application [31].

## 3. Parkinson’s Disease and Vitamin D

Parkinson’s disease (PD) is a progressive neurodegenerative disease characterized by slow, selective dopaminergic neuronal loss. Symptoms include dyskinesia, rigidity, and tremor, as well as postural instability and gait disorders [133]. Like for MS, vitamin D could play a key role in neurological disorders such as PD and dementia [8,134].

In the context of PD, potential neuroprotective effects exerted by vitamin D include the notion that 1,25(OH)2D indirectly inhibits the synthesis of nitric oxide, a free radical that can damage cells [6]; secondly, it indirectly stimulates the synthesis of the antioxidant glutathione [6], and; thirdly, vitamin D may act as a neurotrophic factor, through the stimulation of nerve growth factor (NGF), glial cell line-derived neurotrophic factor (GDNF) and neurotrophin 3 (NT3) [135,136,137]. 

Cross-sectional studies have linked vitamin D deficiency and PD incidence [138,139]. The first longitudinal study investigating the association between vitamin D status and risk of PD showed that low serum vitamin D levels predicted an elevated risk of PD [140], but such findings were not reproduced in a study published later [141]. A recent study by Fullard et al. evaluated vitamin D levels in a population at risk for developing PD, derived from The Parkinson Associated Risk Syndrome (PARS) study cohort. In particular, they found that vitamin D levels did not differ in the high-risk group when compared with age- and sex-matched controls, suggesting that sustained vitamin D insufficiency is not common before a diagnosis of PD [142]. This is in line with another recent study of Larsson et al., which used Mendelian randomization approach to minimize confounding effects and prevent bias because of reverse causation. According to recent findings, their results showed no association between genetically-predicted lower vitamin D concentration and PD [143].

In patients with overt PD, however, it has been repeatedly demonstrated that serum 25(OH)D is significantly lower than in healthy controls [139,144,145,146,147,148,149], and serum 25(OH)D levels progressively decrease with increasing severity of PD [145,150,151]. Intuitively, this link could relate to the reduced sun exposure, hence the dermal synthesis of vitamin D, in patients with progressive motor limitations, whereas no significant difference in vitamin D intake was found in patients versus controls [152]. When the relationship between serum 25(OH)D level and functional scores such as balance is accounted for, a study by Peterson et al. investigated this association using five tests (i.e., the motor control test, sensory organization test, sit and stand test, walk and turn test, and the unilateral stance eyes open and closed test) in 40 PD patients and showed a significant positive correlation between serum 25(OH)D and automatic postural responses [153].

Interventional studies examined the correlation between vitamin D supplementation and PD outcomes. In particular, Suzuki and colleagues [154] examined the impact of vitamin D supplementation (1200 IU per day for one year) on disease progression using various PD-related outcomes measured with the modified Hoehn & Yahr (H&Y) scale and the Unified Parkinson Disease Rating Scale (UPDRS). In a double-blind placebo-controlled trial on 104 patients with PD studied for 12 months, 56 PD patients received 1200 IU vitamin D per day and 58 received placebo. The authors found that those on placebo experienced worsening PD outcomes, while the deterioration was significantly milder in the vitamin D-supplemented group. Analysis of the VDR single nucleotide polymorphisms (SNPs) in these patients found that those bearing the FokI VDR genotype (rs10735810) TT allele had a more significant and consistent response to vitamin D supplementation than individuals with VDR FokI CT, while patients with FokI CC showed no significant effect of vitamin D supplementation compared to placebo. Alternatively, the effects of vitamin D were not influenced by other VDR variants, that is, rs1544410 (BsmI), rs731236 (TaqI), rs7975232 (ApaI), and rs11568820 (Cdx2) [154]. The role of other VDR polymorphisms has also been investigated in PD [153,155,156,157,158,159]. The rs10735810 (FokI) C allele was present at significantly higher frequency in PD patients as compared to controls [156,157]. In a study by Suzuki et al. in a Japanese population, the VDR variant FokI CC was associated with milder forms of PD [158]. Tanaka et al. found a significant inverse association between VDR SNP rs2228570 and the risk of PD, but this fell below significance after adjustment for multiple comparisons [159].

Based on the previous, additional studies are warranted to elucidate the effects of vitamin D on neuroprotection in PD.

## 4. Alzheimer’s Disease and Vitamin D

Alzheimer’s disease (AD) is a neurodegenerative disorder characterized by progressive and irreversible cognitive deficits and behavioural alterations. Memory impairment and loss of spatial memory are hallmarks of the disease, and lead to complete incapacity and death within three to nine years since diagnosis [160]. At pathology, AD presents with amyloid-β (Aβ)-rich plaques, neurofibrillary tangles (NFTs), synapse loss, and atrophy in brain areas associated with memory and executive functions [161,162,163,164]. Aging is a strong risk factor for AD [165,166]. Other major risk factors for AD include gender, family history, genetics, and head trauma, low educational attainment and environmental factors. There is accumulating evidence suggesting a significant association between vitamin D and AD [167,168,169,170]. In vitro, vitamin D stimulates macrophages which increase the clearance of Aβ plaques [171,172], reduces amyloid-induced cytotoxicity and apoptosis in primary cortical neurons [173], and influences Aβ stimulation of induced nitric oxide synthase (iNOS), which contributes to modulate the inflammatory process related to AD [174]. Recent genome-wide association studies have focused on the role of VDR polymorphism in late onset AD (LOAD) susceptibility [175]. A decreased level of VDR mRNA has been reported in hippocampal region by analyzing postmortem AD brain [176]. Alterations in VDR and 1,25-*MARRS* genes related to the action and metabolism of vitamin D result in the inefficient utilization of vitamin D, making neurons vulnerable to neurodegenerative changes [173,174,175,177,178,179]. Associations have been found between AD, VDR gene polymorphisms and megalin to support this inference [174,175,177,179,180,181]. Recently, Gezen-Ak et al. focused on the creation of a condition that prevents the genomic or non-genomic actions of vitamin D by individually or collectively silencing the VDRs (VDR or protein disulfide isomerase A3 (PDIA3)/1,25-*MARRS*) in primary cortical neurons [182]. Using this experimental model, an effect of VDRs disruption could be documented on proteins involved in secretases relating to amyloid pathology and Aβ 1−42 production [182]. The authors then suggested that vitamin D and its receptors VDR and PDIA3 could play important roles in amyloid processing pathway within neurons [182]. The vitamin D carrier, DBP, was also identified at decreased levels upon plasma protein profiling of subjects with mild cognitive impairment and in AD patients, when compared to healthy controls [183]. In a study by Moon et al., the DBP was found to inhibit Aβ aggregation and prevent Aβ mediated cell death in cultured hippocampal cells [184]. Both monomeric and oligomeric Aβ bound to DBP in a dose-dependent manner. Following addition of Aβ, DBP treatment resulted in reduced synapse loss in mouse hippocampus and rescued Aβ-induced memory deficits [184]. Collectively, these results suggest that impairment in vitamin D transport is present even at early stages of AD, namely, prior to development of dementia. Based on this evidence, it is feasible to hypothesize that AD might be the consequence of a hormonal imbalance in which the critical hormone is vitamin D. Vitamin D promotes neuroprotection in AD via regulating nerve growth factor and neurotransmitters [185,186], increasing the amyloid metabolism [187,188], imposing an anti-inflammatory action [189,190] and promoting calcium homeostasis [191]. With this emerging evidence, vitamin D has important roles as a neurosteroid in AD.

Epidemiological studies outlined a strong correlation between deficiency of vitamin D and neurodegeneration associated with AD. As many as 70–90% AD patients are vitamin D deficient, and AD patients are the most vulnerable group to develop neurodegenerative disorder due to additive effect of Vitamin D deficiency along with aging factor [187,192,193,194]. Although vitamin D status is a crucial but non-specific risk factor for AD [195], Cui et al. suggested that specific “critical windows” may exist during which vitamin D deficiency might result in the most detrimental brain outcomes. During these timeframes, vitamin D supplementation could be the most beneficial factor to prevent long-term damage to the brain [26]. A potential therapeutic window during which vitamin D might provide benefits to reduce the risk, or delay the onset, of AD could occur during the pre-clinical and mild cognitive impairment ages, when measurable changes in glucose utilization and Aβ accumulation already occur [196]. 

Llewellyn et al. showed an increased risk of losing points on the Mini-Mental State Examination (MMSE) in six years in 175 older adults with severe 25(OH)D deficiency (<10 ng/mL) compared to 157 subjects with sufficient vitamin D (>30 ng/mL) [192,197]. Slinin et al. followed up the association between lower 25(OH)D levels and cognitive decline in aged individuals (>65 years) for four years [192,198]. A meta-analysis by Etgen et al. highlighted an increased risk of cognitive impairment in patients with vitamin D deficiency [199]. Balion et al. compared mean MMSE scores with levels of 25(OH)D and showed higher average MMSE scores in those with higher 25(OH)D concentrations [200]. Further, in the Chianti study, a large prospective study on 858 adults, cognitive decline was associated with lower concentrations of vitamin D, when observed over a period of six years [197]. The association between low vitamin D and increased risk of AD has been confirmed in other long-term studies [201], and over 50% of published prospective studies showed an elevated risk of cognitive impairment after four to seven years of follow-up in participants with lower 25(OH)D levels compared with participants with higher 25(OH)D levels [195]. In parallel, an increased incidence of vitamin D deficiency has been documented in AD patients [192,200], while a seven-year follow-up study by Annweiler et al. found that higher dietary intake of vitamin D was associated with a lower risk of developing AD in older women [201]. Together, these studies suggested that low vitamin D poses a serious risk for AD development and progression. 

Interventional studies using vitamin D in combination with anti-AD drugs have shown fairly encouraging results. A recent six-month trial study by Annweiler et al. observed that the combination of memantine, a multi-target therapy for AD-related dementias (ADRD), and vitamin D elicited a superior effect than memantine or vitamin D alone in halting the cognitive decline among AD patients tested by MMSE [202]. The AD-IDEA trial [203], a randomized placebo-controlled trial initiated in 2011 and completed in 2016, further investigated the effect of this combination therapy and awaits publication [203]. Fiala and Mizwicki [204] added further evidence that combined administration of vitamin D and docosahexaenoic acid (DHA) could enhance direct effects and immune protection of neurons against brain amyloidosis and other brain insults. As vitamin D targets various pathological processes of ADRDs, it may thus increase the effectiveness of standard anti-dementia treatments, or improve at least partially the resistance to these treatments.

Long-term randomized clinical trials of vitamin D supplements in large populations of middle-aged adults from different countries are warranted to study the conversion rate to mild cognitive impairment or AD outcome, with the most obvious question remaining whether optimizing vitamin D status will reduce the risk AD or be of therapeutic benefit following the onset of the disease. 

## 5. Neurocognitive Disease and Vitamin D

Epidemiological studies outlined an association between 25(OH)D and parameters of cognitive function, such as memory, orientation and executive functions [205,206,207]. Hypovitaminosis D is associated with altered domain-specific cognitive and executive functions, particularly information processing speed, mental shifting and working memory [200], whereas the effect on episodic memory is milder [200]. These functions are required for the cognitive control of behavior and the execution of cognitive programs in real time, and are involved in high-level motor control [208]. In older adults living independently, low serum 25(OH)D concentrations were shown to be significantly associated with cognitive impairment [209]. In addition, certain variants of human VDR gene are associate with increased risk of cognitive decline [176,179]. A study by Llewellyn et al. in 1766 older adults from the Health Survey for England 2000 described an inverse association between 25(OH)D levels and cognitive impairment [205], and patients with 25(OH) levels <20 ng/mL harboured a 230% higher risk of cognitive impairment when compared with those having 25(OH)D levels >20 ng/mL. The same group of authors showed that impairment of cognitive function during a three- and six-year follow-up was greater in patients who were initially vitamin D deficient [197]. In fact, cognition scores measured by MMSE and Trail-Making Tests A and B were poorer in vitamin D-deficient subjects as compared to those whose vitamin D levels were sufficient. Having severe vitamin D deficit at baseline increased the likelihood of showing cognitive decline of executive functioning (measured by MMSE and Trail B) but not attention (measured by Trail A) at the six-year follow-up [197]. Similar results have been obtained in a systematic review and meta-analysis [200]. Full evidence of a causal relationship between low vitamin D and cognitive impairment is far to be demonstrated and the existence of this association has even been challenged in other studies [198,200,210,211]. Nevertheless, some authors concluded that vitamin D deficiency in older adults is associated with dementia and that vitamin D supplementation might have a protective effect [201,212,213,214,215]. Importantly, systematic reviews and meta-analyses carry several limitations while cross-sectional studies cannot provide causal links, that is, answer the question of whether vitamin D deficiency leads to cognitive decline or whether people with a cognition disorder have lower exposure to sunlight and/or lower vitamin D intake [216]. A recent systematic review by Sommer et al. evaluated the influence of vitamin D deficiency on dementia risk using longitudinal studies [217], and concluded that available findings were consistent with the hypothesis that low vitamin D levels could contribute to the development of dementia, although methodological issues should be taken in account, such as residual confounders, single vitamin D assessments, and sunlight exposure [217].

Based on these potential links, it appears advisable that normal levels of vitamin D should be attained to confer potential protection against the risk and progression of AD. In older adults and AD patients, dietary vitamin D intake was associated with better cognitive performance [218], and being in the highest quintile of vitamin D dietary intakes was associated with a lower risk of AD after seven years, when compared with the lower four quintiles combined (adjusted OR = 0.23; *p* = 0.007) [201]. This pro-cognition effect was confirmed in before–after and non-randomized clinical trials on vitamin D supplementation, where improved cognitive performance was recorded in the older population as well as AD patients [219,220,221]. Short-term studies underscored the cognitive benefits elicited by four-week vitamin D supplementation [219], with marked improvements in executive functions and information processing speed [222]. The administration of supra-physiological doses, such as 7000 IU/day, seems to provide no additional benefit [221], while common supplementation dosages around 800–1200 IU/day appear to be sufficient and desirable [223]. Because confounding factors could influence cognition outcomes [195], it remains crucial to conduct appropriately designed, randomized, placebo-controlled interventional trials to test the effectiveness of, and responsiveness to, vitamin D supplementation in AD, once the basal level of vitamin D and other possible confounders are appropriately accounted for [13].

## 6. Amyotrophic Lateral Sclerosis and Vitamin D 

Amyotrophic lateral sclerosis (ALS) is a fatal neurodegenerative disease of the upper and lower human motor system, linked to abnormalities in the glutamate neurotransmitter system [224]. With familial ALS accounting for 5–10% of cases, neurodegeneration is a hallmark of ALS and results from the complex interaction between genetic and molecular pathways, encompassing glutamate excitoxicity, generation of free radicals, cytoplasmic protein aggregates, modifications of the superoxide dismutase (SOD1) enzymes activity, mitochondrial dysfunction, accumulation of intracellular calcium, all leading to disruption of axonal transport through accumulation of intracellular aggregates [225,226,227,228,229]. Challenging the “neurocentric” view of ALS, recent evidence suggests that non-neural cells such as microglia, astrocytes, peripheral blood mononuclear cells (PBMCs) and skeletal muscle fibers may partake in motor neuron degeneration and cooperate to exacerbate ALS [230]. Genetic studies have identified several proteins linking vitamin D to ALS pathology: MHC class II molecules; toll-like receptors; poly(ADP-ribose) polymerase-1 and calcium-binding proteins [231]. Genome-wide analysis further identified a number of biologically relevant candidate genes with VDR-binding sites within or in close proximity [232], and it has been shown that candidate genes involved in gene transcription associated with ALS signaling are modulated by 1,25(OH)2D [233]. Altered calcium homeostasis appears to contribute significantly to selective neuronal injury in ALS, with the putative cause of vulnerability being the low levels of the calcium-binding proteins calbindin-D28K and of parvalbumin, which can be elevated by gene therapy and by vitamin D supplementation [231,234,235]. Calbindin-D28K is a 1,25(OH)2D-induced calcium-binding protein, and is stimulated at the protein and gene level by calcitriol in human syncytiotrophoblast cells [236]. Parvalbumin increases in the caudate putamen of rats with vitamin D hypervitaminosis [237], suggesting a link between parvalbumin metabolism in the caudate putamen and vitamin D variations in the bloodstream. Also, the injection of 80–120 ng calcitriol in the cerebral ventricles of adult rats induced positive immunoreactivity for calcium binding proteins in ventral motor neurons [238], implying a potential modulatory effect of calcitriol on the expression of calcium binding proteins in the motor system. In mice expressing ALS-linked mutated SOD1, an animal model of ALS, vitamin D intake increased muscle strength with no significant effect on lifespan [235], whereas vitamin D3-deficient diet delayed disease onset but decreased motor performance [239]. Treatment of rodent motor neurons with 1,25(OH)2D promoted efficient binding and nuclear translocation of the VDR, exerted a neuroprotective effect against the motor neuron-restricted Fas death pathway, and acted by potentiating the trophic activity of neurotrophins [240]. 

Among the other effects of vitamin D on the proteome linked to ALS, vitamin D has been shown to upregulate VEGF by response element in the VEGF promoter, which may delay progression of ALS [241]. In addition, vitamin D increases neurotrophic factors [242], and acts to promote the effectiveness of IGF-I by increasing expression of IGF-I receptors [243], which promotes survival and motor function parameters in SOD1 animals [244]. Further, vitamin D exerts pro-differentiative, immunomodulatory and anti-inflammatory properties (e.g., by reducing tumor necrosis factor (TNF)-α, IL-1B, and cyclooxygenase (COX)-2), which could prove useful to control the expression of pro-inflammatory molecules linked to ALS progression [245,246], as well as to partake in autophagy-based misfolded SOD1 aggregates clearance [247]. A direct association has also been demonstrated between circulating parathyroid hormone (PTH), which increases in parallel with the severity of vitamin D deficiency, and the duration of ALS in males [248]. Finally, the gene encoding DBP (group-specific component, Gc) is a key factor for regulating calcium homeostasis through the vitamin D endocrine system. As such, a proteomic study of Portuguese patients with familial ALS (FALS), not carrying SOD1 mutations, isolated an isoform of DBP that was identified as GC2, which was absent in healthy controls, and noted a decrease of more acidic isoforms of DBP in FALS; collectively, these results suggest that GC2 polymorphism of DBP could constitute a risk factor for ALS [249].

An interaction also links vitamin D to muscle morphology and strength [250]. The VDR is expressed in skeletal muscle and modulates 25(OH)D uptake in myofibers [251] via the cell membrane receptor megalin, and DBP is retained in these cells by its specific binding to actin [252]. Silencing of the VDR in mice is associated with smaller muscle fibres and persistent immature muscle-gene expression during adult life [253]. In C2C12 muscle cells, 25(OH)D and 1,25(OH)2D dose-dependently exert an auto-regulatory loop to regulate the expression of genes related to proliferation, differentiation, and myotube size [254]. Tracer studies and epidemiological investigations have described a relationship between 25(OH)D levels and parameters relating to muscle, such as lean body mass or exercise performance, leading to speculate that skeletal muscle cells could act as a reservoir of 25(OH)D capable of protecting it from liver uptake, degradation and excretion [252]. In their meta-analysis, Stockton et al. suggested that the beneficial effects of vitamin D on lower extremity muscle strength depended on baseline 25(OH)D levels <25 nmol/L, suggesting that vitamin D effects on muscle performance are more evident in individuals with lower initial 25(OH)D levels [255]. Therefore, the association between vitamin D and muscle morphology and function could lead to hypothesize that vitamin D effects on the muscle contribute to ALS-related function scores [256].

These mechanistic associations notwithstanding, conflicting results from clinical studies exist on prevalence rates of vitamin D deficiency and the effect of vitamin D supplementation in ALS. Using mortality data, a geographic distribution of ALS with a northwest to southeast gradient was demonstrated in an American cohort [257], a finding recently mirrored in a study showing higher ALS-associated death rates in more northern states [258]. An earlier study suggested a high prevalence of hypovitaminosis D in ALS patients [259]. A subsequent study investigating vitamin D levels in 37 consecutive ALS patients found that 81% had vitamin levels below the normal range [234]. In ALS patients, severe vitamin D deficiency was associated with four-fold increased rate of functional decline and significantly reduced survival expectancy [240]. In this study, there was an association between low vitamin D and a survival time in ALS patients, even after excluding non-ambulatory patients with vitamin D deficiency. In 71 sporadic ALS patients, 1,25(OH)2D levels were found significantly lower than in controls, with positive correlations between 1,25(OH)2D and both ALS functional rating scale (ALSFRS-R) and Manual Muscle Test scoring (MMT) [260]. Moreover, the levels of 1,25(OH)2D levels were lower in spinal-onset ALS compared to bulbar-onset ALS, and only in spinal-onset ALS 1,25(OH)2D levels positively correlated with functional scores and negatively with disease duration, suggesting that different impairment of vitamin D signaling pathways in cortical/spinal cord and bulbar motoneurons could explain different levels related to ALS site of symptoms onset [260]. However, divergent conclusions were reached by other studies. In a small retrospective study in ALS and controls, lower than expected rates of vitamin D deficiency were observed in ALS, and there was a lack of relationship between vitamin D and clinical variables related to ALS [261]. A prospective study evaluating vitamin D levels in 106 ALS patients, half of whom were on riluzole, found low vitamin D levels in 69% of cases (<30 ng/mL), with 50% falling into the category of “insufficient” 25(OH)D and 19% being “deficient” [256]. In this cohort, higher vitamin D was associated with higher concurrent gross motor ALSFRS-R scores at baseline. However, vitamin D failed to predict the future rate of disease progression, suggesting that low vitamin D may be a result rather than a cause of worse health in people with ALS, perhaps due to poor mobility and/or metabolic confounders [256]. In a study focused on the association of clinical disease outcome with bone metabolism, including serum 25(OH)D concentrations and bone mineral density, Yang et al. found that 34% of Korean ALS patients were severely vitamin D deficient (<10 ng/mL) and 81% were vitamin D deficient (<20 ng/mL); nevertheless, 25(OH)D concentration failed to predict survival, and older age at onset and bulbar onset were the most predictive factors for survival outcome [262]. Emphasizing these contrasting findings even further, Blasco et al. reported that high-range vitamin D levels were correlated with a worse rather than with the improved prognosis in ALS patients [263]. 

The effect of vitamin D supplementation on ALS progression has only been investigated in a few studies to date. Daily supplementation with 2000 IU of vitamin D for nine months to ALS patients improved the revised ALSFRS-R scores, such that 20 of these patients received vitamin D and their functional scores declined less rapidly than those patients who did not receive supplements [234]. Inversely, in their retrospective study Libonati et al. reported that cholecalciferol treatment for three and six months (loading dose of 100,000 IU every week for four weeks, then maintenance dose of 25,000 IU every 15 days) did not improve clinical parameters compared to untreated ALS patients [261].

Based on these results, evidence to support a causative role for low vitamin D in ALS is weak, and low levels of vitamin D found in patients with ALS could depend on limited mobility, hence sun exposure, in ALS patients. The benefits of vitamin D supplementation in ALS patients remain to be elucidated.

## 7. Conclusions

Accumulating evidence suggests that vitamin D acts like a neurosteroid [29] and is required for normal brain development and function [6,7,8,9,10,11,12,29,264]. The association between low levels of 25(OH)D and a wide spectrum of neurodegenerative conditions such as multiple sclerosis, Alzheimer’s disease, Parkinsons’s disease and neurocognitive disorders, is supported by in vitro and in vivo data. Less convincing, on the other hand, appears to be the causative link between vitamin D deficiency and the onset, progression and clinical burden of amyotrophic lateral sclerosis. Regrettably, most studies conducted to date have not controlled for reverse causality, that is, low levels of vitamin D possibly being due to impaired mobility or sun-avoidance behavior. As such, there is a need for randomized clinical trials on vitamin D treatment in patients at risk of neurodegenerative disorders so as to optimize knowledge and precision on the efficiency of vitamin D congeners, appropriate dosing, and correct biochemical and clinical monitoring. A proper endocrine approach would suggest the use of vitamin D congeners related to optimal drug delivery through the brain blood barrier [265], using gradually increasing doses to avoid the risk of falls in frail populations [266]. In view of the advantage that vitamin D supplementation is readily available and affordable, there is a need for further research in this field.
